# *Hydra vulgaris* exhibits day-night variation in behavior and gene expression levels

**DOI:** 10.1186/s40851-019-0127-1

**Published:** 2019-03-08

**Authors:** Hiroyuki J. Kanaya, Yoshitaka Kobayakawa, Taichi Q. Itoh

**Affiliations:** 1 Department of Biology, School of Science, Kyushu University, Fukuoka, 819-0395 Japan; 2 Faculty of Arts and Science, Kyushu University, Fukuoka, 819-0395 Japan

**Keywords:** Day–night variation, Transcriptome analysis, Diel cycle genes, Cnidarians, *Hydra vulgaris*

## Abstract

**Background:**

Day–night behavioral variation is observed in most organisms, and is generally controlled by circadian clocks and/or synchronization to environmental cues. *Hydra* species, which are freshwater cnidarians, are thought to lack the core clock genes that form transcription–translation feedback loops in clock systems. In this study, we examined whether hydras exhibit diel rhythms in terms of behavior and gene expression levels without typical clock genes.

**Results:**

We found that the total behavior of hydras was elevated during the day and decreased at night under a 12-h light–dark cycle. Polyp contraction frequency, one component of behavior, exhibited a clear diel rhythm. However, neither total behavior nor polyp contraction frequency showed rhythmic changes under constant light and constant dark conditions. To identify the genes underlying diel behavior, we performed genome-wide transcriptome analysis of hydras under light–dark cycles. Using three different analytic algorithms, we found that 380 genes showed robust diel oscillations in expression. Some of these genes shared common features with diel cycle genes of other cnidarian species with endogenous clock systems.

**Conclusion:**

Hydras show diel behavioral rhythms under light–dark cycles despite the absence of canonical core clock genes. Given the functions of the genes showing diel oscillations in hydras and the similarities of those genes with the diel cycle genes of other cnidarian species with circadian clocks, it is possible that diel cycle genes play an important role across cnidarian species regardless of the presence or absence of core clock genes under light–dark cycles.

**Electronic supplementary material:**

The online version of this article (10.1186/s40851-019-0127-1) contains supplementary material, which is available to authorized users.

## Background

Many organisms show changes in metabolic, physiological and behavioral states depending on the time of day. These changes are fundamentally controlled by endogenous circadian clocks. Circadian clocks are composed of transcription-translation feedback loops of clock genes [1–3]. Molecular clocks govern the expression of various genes involved in metabolic, physiological and behavioral regulation, leading organisms to alter their status at certain times of day. Circadian clocks can synchronize with various zeitgebers to adjust to the environmental time [4]. Light, one of the strongest zeitgebers, resets the states of organisms via their circadian clocks. Clock systems are conserved from bacteria to humans, and in many organisms, the clock shows a diurnal rhythm under light–dark conditions, adjusts to new environmental conditions, and retains the circadian rhythm even under constant environmental conditions [4, 5].

Cnidarians appeared 740 million years ago and are the sister group to bilaterians [6, 7]. Cnidarians are classified into five main groups: *Anthozoa* (sea anemones and corals), *Scyphozoa* (true jellyfishes), *Cubozoa* (box jellyfishes), *Staurozoa* (stalked jellyfishes) and *Hydrozoa* (a diverse group including freshwater cnidarians) [8, 9]. Cnidarians’ primitive nervous system lacks a conspicuous central nervous system [10, 11]. However, even in cnidarians, day-night variation exists in behavioral and physiological states, and it has been clarified that such diurnal variation accompanies alterations in gene expression [12–17]. In both *Acropora millepora* and *Nematostella vectensis*, which are in the class *Anthozoa*, diel cycle genes showing rhythmic expression under light–dark cycles have been identified by transcriptome analysis [12, 16]. Although *period* and *timeless* are absent in these cnidarians, orthologs of core clock genes such as *Clock*, *Bmal*, and *Cryptochrome* (Type I and II) are conserved and are thought to form transcription–translation feedback loops to act as circadian clocks [18–20].

*Hydra* spp. are small freshwater cnidarians belonging to the class *Hydrozoa* that have been used as model organisms for studies on regeneration, stem cell differentiation, aging, and symbiosis [21]. The *Hydra* genome was decoded in 2010, and interestingly, it was revealed that there were no core clock genes in the genome [22]. Hydras react to light, although it has been found that hydras do not respond to red light [23–26]. For instance, light can induce polyp contraction, a typical behavior in hydras [27–29]. Discharge of cnidocytes is also affected by light [30]. Moreover, hydras exhibit phototaxis and move toward light sources [31, 32]. However, while light is considered to be an important signal affecting the physiological states of hydras, little is known about the diurnal behavior and physiological states of these organisms. In this study, we found that hydra behavior showed day-night variation during a 12-h light–dark cycle (LD 12:12 cycle) by quantifying behavior with a video analysis system. Furthermore, we identified hundreds of genes whose expression showed diel oscillations under LD 12:12 cycles by genome-wide transcriptome analysis. Interestingly, the expression patterns of some of the genes are conserved in other cnidarian species that possess endogenous clock systems. Based on these data, we hypothesized that hydra behavior is affected not only by light–dark cycles but also by diel cycle genes.

## Materials and methods

### Animals

*Hydra vulgaris* (strain 105) without buds were used in all experiments. The hydras were maintained in a hydra culture solution (HCS; 1 mM NaCl, 1 mM CaCl_2_, 0.1 mM KCl, 0.1 mM MgSO_4_, 1 mM tris-(hydroxymethyl)-amino-methane; pH 7.4, adjusted with HCl) at 20 °C under a 12-h light–dark cycle (LD 12:12 cycle). Light intensity was maintained at approximately 450 lx. The hydras were fed with newly hatched Artemia nauplii twice per week.

### Behavioral tracking and data analysis

**Fig. 1:**
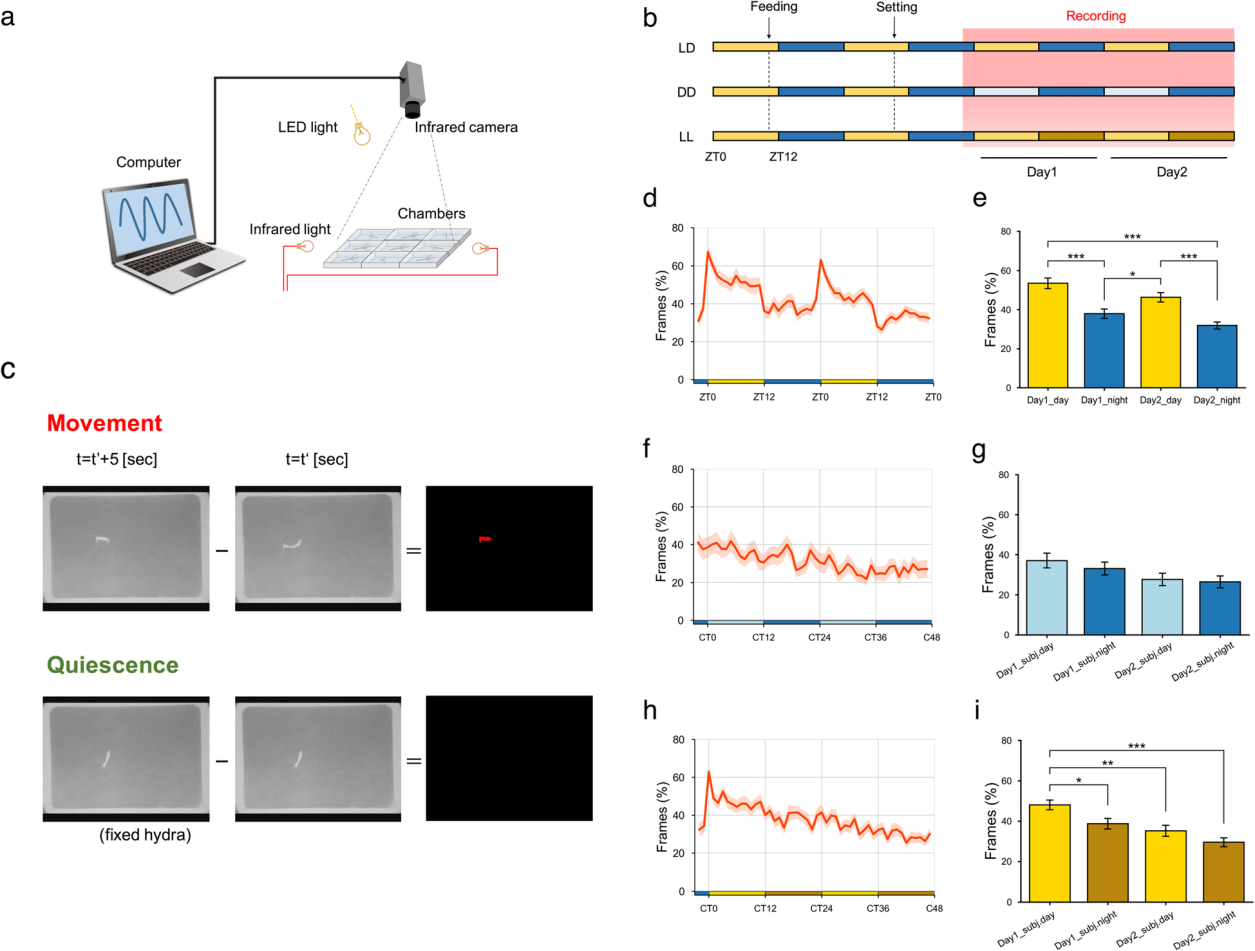
Hydras exhibit behavioral day-night variation **a** Behavioral recording system. Hydras were placed into rectangular chambers and illuminated with infrared light. The movements of the hydras were captured using an infrared camera. **b** Experimental scheme of behavior analysis under LD 12:12 cycles, DD and LL conditions. Hydras fasted for 24 h were placed into the chamber at ZT10 (setting). Light blue indicates subjective day under DD condition and dark yellow indicates subjective night under LL condition. **c** Frame subtraction analysis. Upper panel: movement of a hydra between two frames was detected as red (t = t’ + 5 and t = t’). Lower: no movement was detected when a fixed hydra was captured and analyzed. **d**, **e** Percentage of frames in which movement was detected during daytime and nighttime under LD 12:12 cycles (*n* = 27). **f**, **g** Percentage of frames in which movement was detected under DD condition (*n* = 26). **h**, **i** Percentage of frames in which movement was detected under LL condition (*n* = 27). The orange lines and shaded areas around the lines show the mean and the SEM, respectively. The bars indicate the SEM. *P < 0.05, **P < 0.01, ***P < 0.001, one-way ANOVA, Tukey post-hoc test. *P* > 0.05 for all comparisons in **g** (under DD) using one-way ANOVA. CT means circadian time

Hydras were starved for 36 h prior to behavioral recording. Each hydra was placed into a rectangular silicone container (22 mm × 32 mm × 5 mm) filled with 3.5 ml of HCS at zeitgeber time 10 (ZT10). The hydras were allowed to acclimate to this new container for 12 h. Video recording was initialized, beginning at ZT22. To allow continuous recording of hydra behavior under both light and dark conditions, the hydras were illuminated with infrared light (Sousin Digital, Tokyo, Japan) and visualized using a video camera (Hanwha Q CELLS Japan, Tokyo, Japan) through an infrared high-pass filter (FUJIFILM, Tokyo, Japan). Frames were captured every 5 s at a resolution of 1280 × 720 pixels and saved with 8-bit grayscale resolution (Fig. 1a, b).

A custom ImageJ macro [33] was used for frame subtraction analysis to quantify hydra behavior. The stored frames were divided into hourly datasets consisting of 720 frames each. Then, the differences in grayscale values (256 gradations) between each pair of images for all pixels were calculated, and new subtracted images were produced. To remove the noise from the newly produced images, an optimal threshold was automatically determined after median filtering. When hydras moved between the two frames, the grayscale values of some pixels exceeded the threshold, which was defined as movement. When all pixel values were lower than the threshold, hydras in these frames were defined as quiescent (Fig. 1c). No significant movement was detected when dead hydras fixed with 8% paraformaldehyde were monitored for one hour and analyzed by the macro. Thus, the custom macro was able to automatically distinguish whether hydras moved between frames. To evaluate hydra behavior, the percentage of frames in which movement was detected was calculated.

### Observation of spontaneous polyp contraction

To continuously observe spontaneous polyp contraction, hydras were prepared and their movements were captured over two consecutive days as described above. Polyp contractions were manually identified using ImageJ or Fiji [34], and the number of contractions was quantified.

### Sampling and RNA isolation

Five hydras were collected every four hours during the LD 12:12 cycles, starting at ZT1. Total RNA was isolated from each sample using TRIzol Reagent (Thermo Fisher Scientific, MA, USA) and purified using an SV Total RNA Isolation System (Promega, WI, USA) according to the manufacturer’s instructions. RNA in samples was quantified by an ND-1000 spectrophotometer (NanoDrop Technologies, DE, USA), and the quality was confirmed with an Experion System (Bio-Rad Laboratories, CA, USA). The samples were biologically replicated at each timepoint.

### Transcriptome analysis by microarray

The cRNA was amplified, labeled, and hybridized to a 4 × 44 K custom-made *Hydra* microarray (Agilent Technologies, CA, USA) according to the manufacturer’s instructions. All hybridized microarray slides were scanned by an Agilent scanner. Relative hybridization intensities and background hybridization values were calculated using Agilent Feature Extraction Software (9.5.1.1). The raw signal intensities and flags for each probe were calculated from the hybridization intensities (gProcessedSignal) and spot information (gIsSaturated, etc.) according to the recommended procedures of the Agilent Flag criteria in GeneSpring Software. Raw signal intensities of all samples were log_2_-transformed and normalized by quantile algorithm with the preprocessCore library package [35] in Bioconductor software [36].

### Analysis of rhythmic gene expression

Genes with rhythmic expression patterns were identified using ARSER [37], JTK_CYCLE [38] and empirical JTK_CYCLE [39]. Log_2_-transformed and normalized data from two daily cycles were used as input data for all programs. In ARSER and JTK_CYCLE, *P* < 0.05 was used as the significance threshold, whereas a Bonferroni-adjusted *P* value < 0.05 was used in empirical JTK_CYCLE. The oscillating genes identified by all three algorithms were classified into four clusters by K-means clustering and analyzed for enrichment of gene ontology (GO) terms. After a search was conducted for the human homologue of each gene (e-value < 1 × 10^− 3^), GO enrichment analysis was performed for each cluster using the Database for Annotation, Visualization, and Integrated Discovery (DAVID) version 6.8 [40]. The human homologues of all the unique genes on the custom-made microarray were used as a background model.

## Results

### Hydras show diurnal behavioral rhythms

To investigate whether hydras show day-night variation in total behavior under a 12-h light–dark cycle (LD 12:12 cycle), we recorded hydra behavior for two consecutive days. Since hydras are not able to respond behaviorally to red light [23], we captured video with infrared illumination to visualize behavior even under dark conditions (Fig. 1a, b). Through frame subtraction analysis of images acquired every five seconds, we detected the frames in which hydras moved (Fig. 1c, see Materials and Methods). Based on the proportion of frames in which motion was detected among the 720 frames acquired in one hour, hydras exhibited marked day-night variation in behavior (Fig. 1d). On the first day, movement was detected in 53.5 ± 2.7% (mean ± SEM) of the total frames during the daytime (ZT0-ZT12) but only in 37.9 ± 2.4% of the total frames during the nighttime (ZT12-ZT24). On the second day, movement was detected in 46.3 ± 2.4% of the total frames from ZT0-ZT12 but only in 31.9 ± 1.8% of the total frames from ZT12-ZT24. These results suggest that hydras are more active in the daytime than in the nighttime under LD 12:12 cycles (Fig. 1e).

To know whether the behavioral variation remains under constant environmental conditions, we recorded hydra behavior under constant dark (DD) and light (LL) conditions (Fig. 1b). Unlike LD 12:12 cycles, there was no circadian variation in hydra behavior under DD condition (Fig. 1f, g.). Under LL condition, although the behavioral onset was induced by lights-on stimulus as well as LD cycles, there was no circadian variation throughout two days (Fig. 1h, i). These results suggest that the behavioral onsets and the offsets are triggered by lights-on and lights-off stimulus, respectively. In other words, the day-night variation of total behavior is induced by the light–dark cycles.

**Fig. 2:**
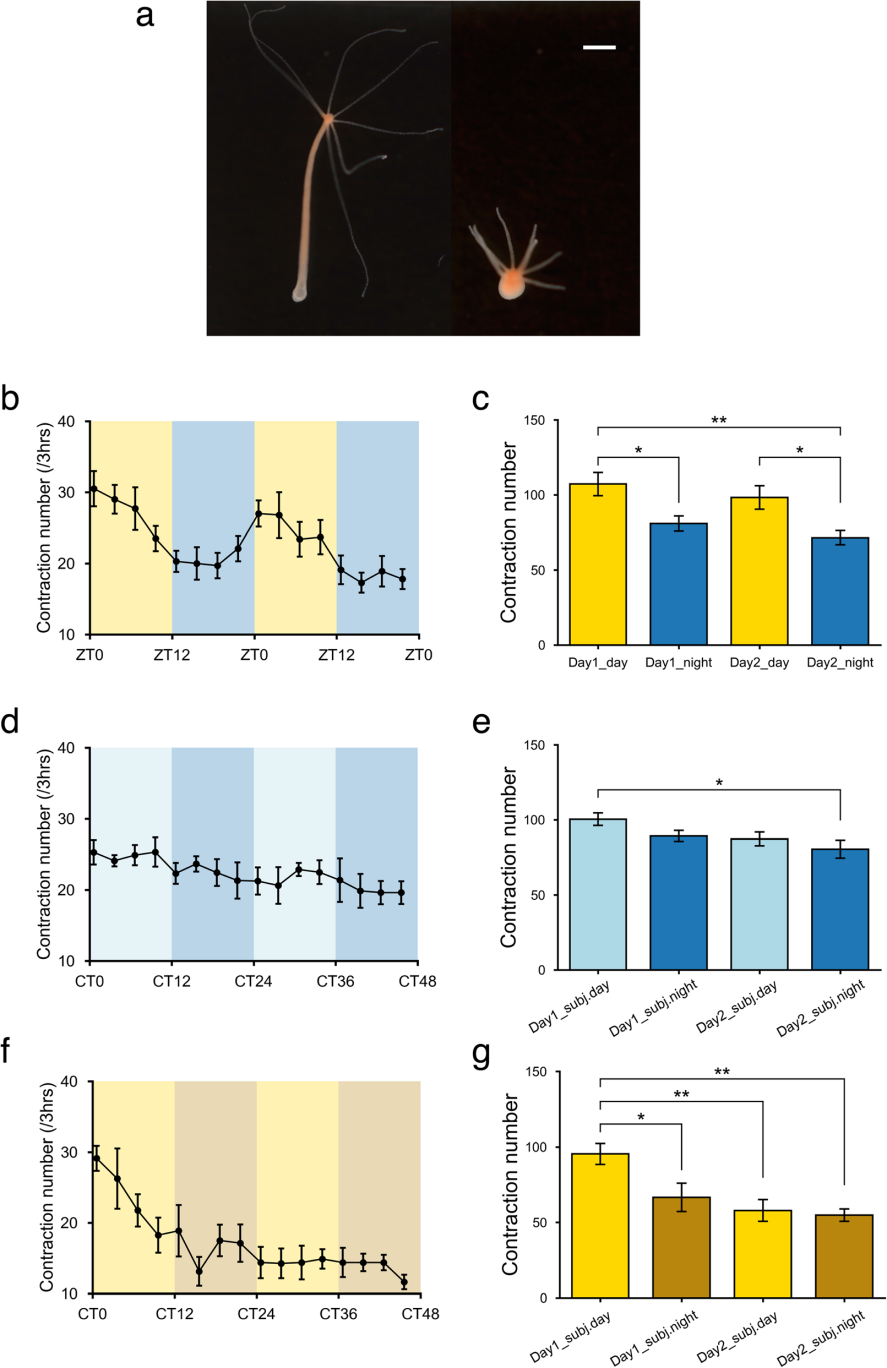
Diurnal rhythm of spontaneous polyp contraction **a** Elongation (left) and contraction (right) of hydra polyps. The white bar indicates 1 mm. **b**, **c** Spontaneous polyp contraction under LD 12:12 cycles (*n* = 10). **d**, **e** Spontaneous polyp contraction under DD condition (*n* = 9). **f**, **g** Spontaneous polyp contraction under LL condition (*n* = 9). The data are shown as the mean ± SEM. *P < 0.05, **P < 0.01, ***P < 0.001, one-way ANOVA, Tukey post-hoc test

Polyp contraction is a typical behavior in cnidarians, including hydras, and appears to be related to osmoregulation and respiration [41–43] (Fig. 2a). Therefore, we next focused on whether polyp contraction showed a diurnal rhythm under LD 12:12 cycles. We manually counted the contractions per three hours over two consecutive days, and the number of contractions clearly showed a diurnal rhythm under the LD 12:12 cycle (Fig. 2b). There were 107.2 ± 7.7 (mean ± SEM) contractions in the daytime (ZT0-ZT12) and 80.9 ± 5.0 contractions in the nighttime (ZT12-ZT24) on the first day, and on the second day, 98.2 ± 7.8 and 71.5 ± 4.7 contractions were detected during the daytime and nighttime, respectively (Fig. 2c). The number of daytime contractions was significantly greater than that of nighttime contractions (Fig. 2c). As well as the total behavior, no circadian rhythm was observed in DD condition although the number of contractions was slightly decreased between the subjective day of the first day and the subjective night of the second day (Fig. 2d, e). Under LL condition, the number of contractions in CT0-CT12 showed the similar pattern as in ZT0-ZT12 of LD cycles, however it kept constant thereafter (Fig. 2f, g). These results suggest that hydras exhibit day-night variation under the light–dark cycles, in both total movements and spontaneous polyp contractions.

### Genes showing diel expression under LD 12:12 cycles

**Fig. 3:**
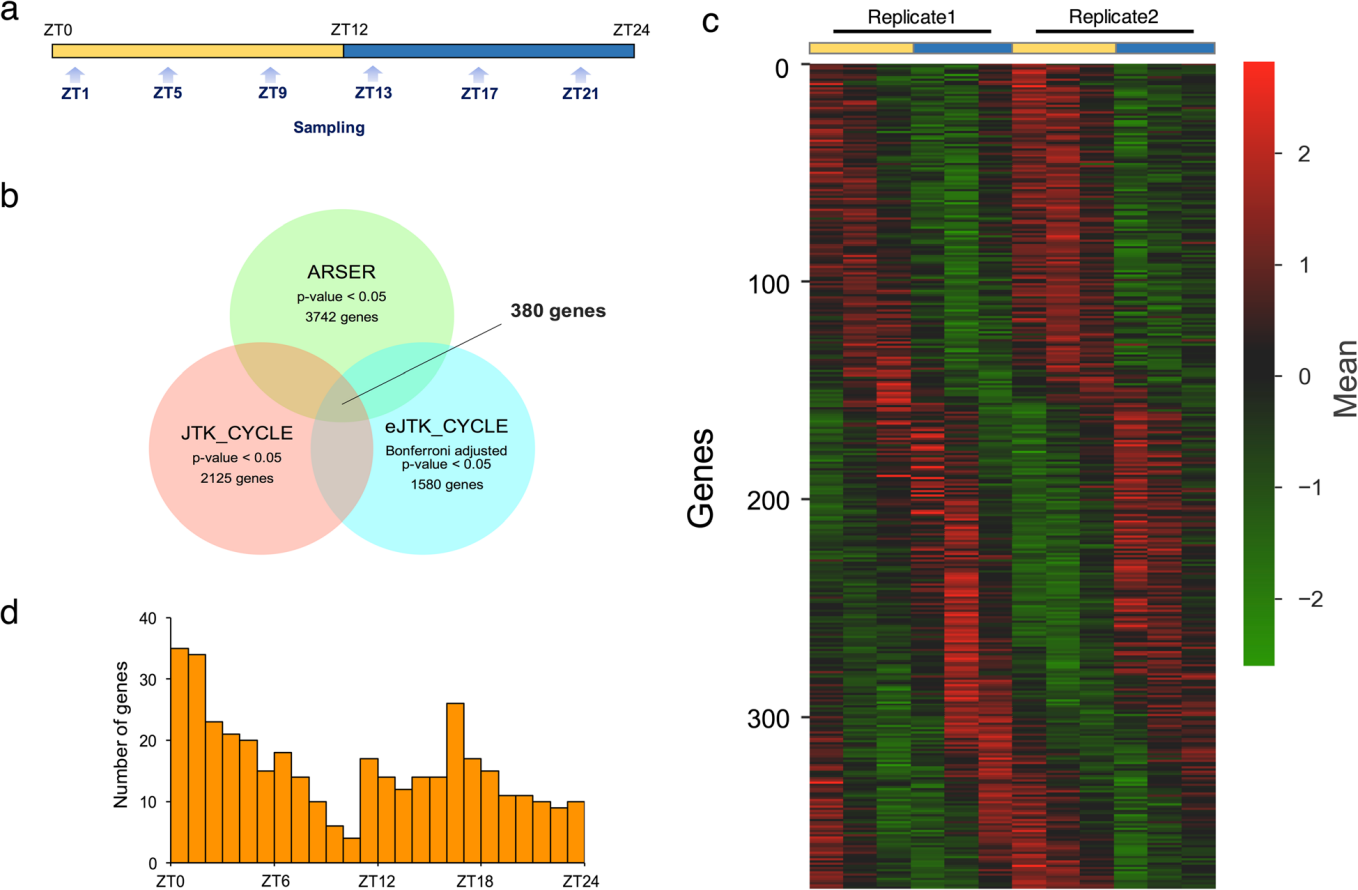
Diel cycle genes in hydras **a** Experimental design for microarray analysis. Hydras were collected every 4 h under LD 12:12 cycles. **b** Detection of diel cycle genes by three algorithms. *P* values were used for the ARSER and JTK_CYCLE algorithms as cutoff thresholds, while Bonferroni-adjusted *P* values were used for the empirical JTK_CYCLE algorithm. The 380 genes identified by all three algorithms were defined as *Hy*DCGs. **c** Phase-sorted heat map of *Hy*DCGs. The data for each gene were normalized so that the mean signal intensity for 12 time points and the standard deviation were 0.0 and 1.0, respectively. **d** Distribution of *Hy*DCGs across phases for a single cosine model (ARSER)

To comprehensively identify the genes related to day-night variation in hydras, transcriptome analysis by microarray was carried out. Beginning at ZT1, we sampled hydras at six time points (every four hours), and gene expression levels were examined with a 4 × 44 K custom-made *Hydra* microarray (Fig. 3a). To evaluate rhythmic gene expression within a circadian period, the expression levels were analyzed with three algorithms, ARSER, JTK_CYCLE and empirical JTK_CYCLE, that are commonly used in rhythmicity detection analysis [44, 45]. Among the 23,903 unique genes, 3742 genes, corresponding to 15.7% of the total number of unique genes on the custom-made microarray, showed significant rhythmicity of expression (*P* < 0.05) by ARSER. On the other hand, 2125 genes (adjusted *P* value < 0.05) were identified by JTK_CYCLE and 1580 genes (Bonferroni-adjusted *P* value < 0.05) were identified by empirical JTK_CYCLE to have significant rhythmic expression patterns. To identify the most reliable rhythmically expressed genes, we extracted the common genes detected by all three analytic algorithms (Fig. 3b). A total of 380 shared rhythmically expressed genes, corresponding to 1.6% of the total number of unique genes, were defined as *Hydra* diel cycle genes (*Hy*DCGs) (Fig. 3c, see Additional file 1). Next, to determine when rhythmic transcription occurred during a day, we examined the peak phases of expression for the *Hy*DCGs. The peak phase distribution of the *Hy*DCGs was observed at approximately ZT0-ZT2 (Fig. 3d). Even in the dark phase, there was a minor peak near ZT17. Given the features of the phase distribution, the *Hy*DCGs may be regulated in multiple ways under LD 12:12 cycles.

### Clustering and gene ontology (GO) enrichment analysis of the diel cycle genes

**Fig. 4:**
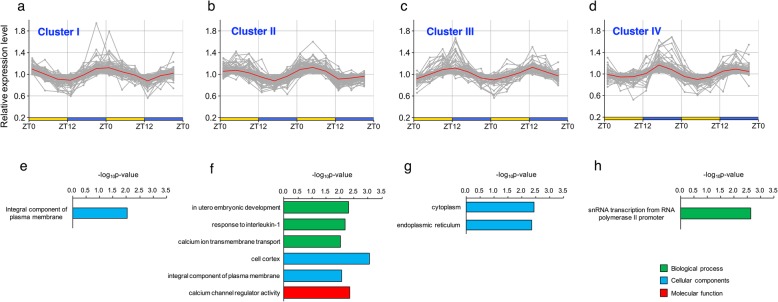
Characterization of diel cycle genes **a**, **b**, **c**, **d** Oscillation patterns the clusters identified by K-means clustering. The red line indicates the mean intensity of the clustered genes. **e**, **f**, **g**, **h** GO terms enriched in Cluster I, Cluster II, Cluster III, and Cluster IV. The *p*-values represent the modified Fischer exact *P* values

To characterize the *Hy*DCGs, we first classified them into four clusters by K-means clustering. Interestingly, the characteristic peak times for the clusters were distributed approximately every six hours. The peak phases of Cluster I (82 genes), Cluster II (133 genes), Cluster III (67 genes) and Cluster IV (98 genes) were near ZT0 (dawn), ZT6 (midday), ZT12 (dusk) and ZT18 (midnight), respectively (Fig. 4a-d). We then performed GO enrichment analysis for each cluster (Fig. 4e-h, see Materials and Methods). Cluster I and Cluster II, which had expression peaks during the daytime, contained genes significantly associated with the ‘integral component of the plasma membrane’ and ‘cell cortex’ terms (Fig. 4e, f). In addition, the GO terms ‘calcium ion transmembrane transport’ and ‘calcium channel regulator activity’ were enriched in Cluster II (Fig. 4f). The GO terms ‘cytoplasm’ and ‘endoplasmic reticulum’ were enriched in Cluster III, while the GO term ‘snRNA transcription from RNA polymerase II promoter’ was enriched in Cluster IV (Fig. 4g, h). These results suggest that some specific cellular functions are performed at a specific time of day.

### Diel cycle genes involved in neuron activity

Similar to the finding that the GO terms ‘integral component of plasma membrane’ and ‘cell cortex’ were enriched in Cluster I and Cluster II, various channel genes and receptor genes exhibited a diel expression pattern. Such oscillations were observed in the expression of four putative potassium channel genes: a calcium channel gene, a nonselective cation channel gene, and two acid-sensing ion channel genes (Table 1). The peak phases of all channel genes occurred during the daytime (ZT1 or ZT5). We also found that several putative neurotransmitter receptors displayed diel expression patterns (Table 2). For example, putative neuronal and muscarinic acetylcholine receptors (Hydra 2.0 Genome Gene IDs: Sc4wPfr_850.1.g5705.t1 and Sc4wPfr_412.g24374.t1, respectively) exhibited this expression pattern, as did a putative GABA receptor gene (Gene ID: Sc4wPfr_1272.g4995.t1) involved in the modulation of pacemaker activity in hydras [46]. Moreover, a homologous gene of Neurexin-3 displayed a diurnal oscillation pattern with peak expression at ZT1. Neurexins participate in synaptic cell-cell adhesion and mediate signaling across synapses [47, 48].

**Table 1:** Putative channel genes exhibiting diel expression patterns

Gene ID	Homologue identified by BLAST2GO	Peak
Sc4wPfr_875.1.g17134.t1	Potassium voltage-gated channel subfamily G member 2 (*R. norvegicus*)	ZT1
Sc4wPfr_698.2.g23056.t1	Endosomal lysosomal potassium channel TMEM175 (*D. rerio*)	ZT1
Sc4wPfr_875.1.g17150.t1	Calcium-gated potassium channel (*T. volcanium*)	ZT1
Sc4wPfr_1100.g15608.t1	Potassium voltage-gated channel subfamily A member (*O. mykiss*)	ZT5
Sc4wPfr_1120.g5076.t1	Voltage-dependent calcium channel subunit alpha-2 delta-3 (*H. sapiens*)	ZT1
Sc4wPfr_615.1.g17637.t2	Transient receptor potential cation channel subfamily A member 1 homologue (*C. elegans*)	ZT1
Sc4wPfr_824.g11302.t1	Acid-sensing ion channel 1 (*R. norvegicus*)	ZT5
Sc4wPfr_2074.g13182.t1	Acid-sensing ion channel 3 (*H. sapiens*)	ZT5

The Gene IDs and the homologues represent the gene numbers and annotations, respectively, registered in [65]. The peak indicates the phase of maximal expression among six time points (ZT1, ZT5, ZT9, ZT13, ZT17 and ZT21)

**Table 2:** Putative neurotransmitter receptor genes exhibiting diel expression patterns

Gene ID	Homologue identified by BLAST2GO	Peak
Sc4wPfr_850.1.g5705.t1	Neuronal acetylcholine receptor subunit alpha-6 (*M. musculus*)	ZT1
Sc4wPfr_1272.g4995.t1	Gamma-aminobutyric acid receptor subunit beta-3 (*R. norvegicus*)	ZT1
Sc4wPfr_719.g8314.t1	Metabotropic glutamate receptor 8 (*M. musculus*)	ZT1
Sc4wPfr_65.g31692.t1	Adenosine receptor A2a (*C. l. familiaris*)	ZT5
Sc4wPfr_412.g24374.t1	Muscarinic acetylcholine receptor M3 (*M. musculus*)	ZT21

The Gene IDs and homologues are based on information registered in [65]. The peak indicates the peak phase of gene expression among six time points (ZT1, ZT5, ZT9, ZT13, ZT17 and ZT21)

Homologous genes of kinesin motor proteins (Gene IDs: Sc4wPfr_1040.g7652.t1 and Sc4wPfr_1402.g13898.t2) and cyclin-dependent kinase-like 5 (Gene ID: Sc4wPfr_126.2.g29250.t2) also showed diurnal expression rhythms. Most kinesins contribute to anterograde transport by moving along microtubule filaments to the plus end [49, 50]. In neurons, kinesin motors are critical for axonal transport of translated proteins and synaptic vesicle precursors [51]. Cyclin-dependent kinase-like 5 not only participates in neural development but also controls axonal transport via phosphorylation of motor proteins [52–54]. The diel expression of these channels, neurotransmitter receptors, and axonal transport-associated genes may reflect day–night variation in neuron activity in hydras.

### RNA processing-associated diel cycle genes

In Cluster IV, whose peak was at approximately midnight, the GO term ‘snRNA transcription from RNA polymerase II promoter’ was enriched (Fig. 4h). Small nuclear RNAs (snRNAs) participate in pre-mRNA splicing by forming small nuclear ribonucleoprotein (snRNP) complexes [55, 56]. Furthermore, we found three homologues of RNA processing-associated genes that exhibited diurnal oscillation, two of which showed peak expression during the nighttime (Table 3). Such expression oscillation might be due to posttranscriptional control and transcriptional regulation during the daytime and nighttime in hydras.

**Table 3:** RNA processing-associated genes exhibiting a diel expression pattern

Gene ID	Homologue identified by BLAST2GO	Peak
Sc4wPfr_629.g5413.t1	Tuftelin-interacting protein 11 (*X. laevis*)	ZT5
Sc4wPfr_886.g29527.t1	Serine/arginine-rich splicing factor 4 (*M. musculus*)	ZT17
Sc4wPfr_423.g13471.t1	Heterogeneous nuclear ribonucleoprotein 1 (*A. thaliana*)	ZT17

The Gene IDs and homologues are based on information registered in [65]. The peak indicates the peak phase of gene expression among six time points (ZT1, ZT5, ZT9, ZT13, ZT17 and ZT21)

### Comparative analysis of diel cycle genes in hydras and other cnidarians

We compared *Hy*DCGs with circadian genes in other cnidarians. In *Acropora millepora* and *Nematostella vectensis,* both of which belong to the class *Anthozoa*, diel cycle genes have been identified by transcriptome analysis using samples collected every four hours [12, 16]. *Acropora millepora* is a kind of coral that harbors endosymbiotic algae, while *Nematostella vectensis* is a nonsymbiotic sea anemone inhabiting the sand of brackish areas. Despite some differences in lifestyle, the two species share some diel cycle genes [16]. Among nine genes, including two types of *Cryptochrome,* that are commonly listed among the clock-controlled genes of *Acropora millepora* and the diel cycle genes of *Nematostella vectensis*, four genes, the transcription factor *Hes1*, a heme-binding protein, a protein disulfide isomerase and heat shock protein 70 kDa, showed diel expression patterns with different peak times in hydras (Table 4). Furthermore, four homologous genes displayed diel oscillation in both *Hydra vulgaris* and *Nematostella vectensis* but not in *Acropora millepora* (Table 5). We also found that three types of collagen genes exhibited diel oscillation in hydras, as has also been shown in *Nematostella vectensis* [16]. On the other hand, some homologues of the RNA processing-associated genes described above (serine/arginine-rich splicing factors) are expressed with a diel cycle in *Acropora millepora* but not in *Nematostella vectensis* [12]. These comparative analysis results suggest that *Hydra vulgaris* shares an analogous profile of diel gene expression with other cnidarians regardless of the presence or absence of canonical clock genes.

**Table 4:** Homologous pairs of diel cycle genes in three cnidarian species (*Hydra vulgaris*, *Nematostella vectensis* and *Acropora millepora*)

Gene ID	Homologue identified by BLAST2GO	Peak	NV_Gene ID	NV_Peak	AM_Peak
Sc4wPfr_372.g27977.t1	Heat shock 70 kDa protein 12A (*H. sapiens*)	ZT1	216,823	ZT17	ZT10
Sc4wPfr_484.g18296.t1	Heme-binding protein 2 (*M. musculus*)	ZT13	114,661	ZT9	ZT6
Sc4wPfr_588.g23926.t1	Protein disulfide isomerase A5 (*M. musculus*)	ZT17	193,399	ZT17	Z0
Sc4wPfr_338.1.g31632.t1	Transcription factor HES-1-B (*X. laevis*)	ZT21	246,249	ZT5	ZT2

The Gene IDs, homologues, and peaks represent the gene numbers, annotations, and peak phases of gene expression in hydras, respectively. NV_Gene ID denotes the gene number for *Nematostella vectensis* registered in [66, 67]. NV_Peak and AM_Peak denote the peak phases of gene expression in *Nematostella vectensis* and *Acropora millepora*, respectively. The peak phase information was derived from previously published data [16]

**Table 5:** Homologous diel cycle genes present in both *Hydra vulgaris* and *Nematostella vectensis* but not in *Acropora millepora*

Gene ID	Homologue identified by BLAST2GO	Peak	NV_Gene ID	NV_Peak
Sc4wPfr_165.g10076.t1	Dual serine/threonine and tyrosine protein kinase (*R. norvegicus*)	ZT9	241,055	ZT5
Sc4wPfr_396.g3020.t1	No hits	ZT13	241,935	ZT1
Sc4wPfr_786.g1715.t1	Elongation of very long chain fatty acids protein (*H. sapiens*)	ZT17	231,850	ZT5
Sc4wPfr_1214.g29727.t3	Serine/threonine-protein phosphatase 6 catalytic subunit (*R. norvegicus*)	ZT17	177,129	ZT5

The Gene IDs, homologues, and peaks represent the gene numbers, annotations, and peak phases of gene expression in hydras, respectively. NV_Gene ID and NV_Peak denote the gene number for *Nematostella vectensis* registered in [66, 67] and the peak expression time for *Nematostella vectensis* [16], respectively

## Discussion

### Hydra behavior can entrain to light–dark cycles

In this study, we clarified that day-night variation exists in hydra behavior. Based on our behavioral recordings, hydras show high behavioral activity in the daytime and less activity in the nighttime (Fig. 1d, e). Although hydras exhibit stable behavior repertories [57], we found that the behavior of hydras entrained to the light–dark cycles. However, the activity pattern was not similar to typical circadian rhythms shown under LD 12:12 cycles because there was a lack of anticipatory behavior prior to the environmental cues (Fig. 1d). If organisms possess endogenous circadian clocks, they are able to show anticipatory behavior, one of the important features of circadian rhythms [58]. As shown in Fig. 1d, hydra activity increased rapidly at lights-on (ZT0) and then slightly decreased until approximately midday (ZT6). From midday to lights-off (ZT12), the activity level was almost constant, but at lights-off, the level dramatically decreased and then remained constant until the end of the night (ZT24). Given that the various light-stimulated behaviors of hydras reported so far started at ZT0 and ceased at lights-off, the day-night variation might be produced by the light-on/off stimulus itself. In fact, hydra activity did not increase at CT0 under DD condition and kept constant throughout two days without circadian oscillations (Fig. 1 f, g). Moreover, since CT0-CT12 under LL is (the) same condition as ZT0-ZT12 under LD cycles, it is unsurprising that these activity patterns were similar to each other; however, there were neither a rapid decrease at CT12, nor circadian rhythms in hydra activity under LL (Fig. 1 h, i). These behavior patterns we observed thus do not contradict previous reports that there are no canonical clock genes in the *Hydra* genome [22]. However, upon separate analysis of polyp contraction behavior, a typical behavior of hydras, it seems that the frequency of contraction may be regulated by endogenous oscillation systems (Fig. 2b). Contraction frequency initially increased at lights-on but then gradually decreased without showing any response to the lights-off stimulus, as shown in Fig. 2b. Then, the frequency mildly increased slightly before the next lights-on stimulus. This pattern closely resembles anticipatory activity, which can be shown in organisms with endogenous circadian clocks [58]. Nevertheless, the frequency of contraction did not show circadian rhythms under DD and LL (Fig. 2d, f). These findings suggest that hydras may possess a circadian system which is only driven by light–dark cycles to regulate polyp contraction behavior even though there are no canonical clock genes in the hydra genome. Although we have clearly shown in this study that hydra behavior at least entrains to LD 12:12 cycles, further studies are necessary to address whether hydras possess endogenous circadian systems for specific behaviors.

### Day–night variation in gene expression levels

Through genome-wide transcriptome analysis, we comprehensively investigated gene expression in hydras under LD 12:12 cycles. This analysis allowed us to identify genes showing diel expression patterns in hydras. *Hy*DCGs corresponding to 1.6% of the total number of genes exhibited striking diel expression patterns. Because we used three analytic algorithms to detect genes with diel oscillation and extracted only the common genes, it is probable that there are more diel cycle genes in hydras than we identified. By K-means clustering, the *Hy*DCGs were classified into four groups with distinct peak phases: dawn, midday, dusk and midnight. This finding suggests that the diel expression patterns are not controlled solely by lights-on or lights-off stimuli. However, considering that the number of genes in each cluster was not evenly distributed (*P* < 0.01, chi-square test), hydras might have a specific time of day where rhythmic transcriptions are actively performed to induce physiological day-night variations.

The *Hy*DCGs included several channels, neurotransmitter receptors, and axonal transport-associated genes. Thus, it is possible that neuron activity changes during the daytime and nighttime in hydras. Neuropeptides are important for the regulation of neuron activity in hydras [59], and recent genome sequencing has revealed that hydras have various neurotransmitter receptors [22, 60]. Previous pharmacological and electrophysiological studies have suggested that several neurotransmitters regulate pacemaker activity involved in spontaneous contraction and elongation in hydras [46, 61]. The diel expression patterns of neuron-associated genes observed in this study may contribute to transitions in behavior.

The diel cycle genes of hydras share common properties with the diel cycle genes of other cnidarians. Through comparative analysis of the genes of hydras and other cnidarians, we found that four genes commonly showed diel oscillations in expression among three cnidarian species (*Hydra vulgaris*, *Nematostella vectensis* and *Acropora millepora*) (Table 4). In addition, several genes showing diel expression patterns were shared between *Hydra vulgaris* and *Nematostella vectensis* (Table 5) or between *Hydra vulgaris* and *Acropora millepora*. Despite their lack of major clock genes, hydras may have systems to produce diel oscillations in gene expression to enable proper biological functioning at certain times of the day that are similar to the systems of other cnidarians with circadian clocks.

### Absence of canonical clock systems in hydras

Several cnidarians in the class *Anthozoa* possess typical core clock genes and exhibit endogenous circadian rhythms in both behavior and gene expression [12, 16]. Moreover, core clock genes are also encoded in the genome of *Clytia hemisphaerica,* a cnidarian of the class *Hydrozoa* [62]. It is thus probable that the loss of clock genes in hydras was secondary. In addition to lacking a central clock system, hydras also rely on photoreception to trigger behavior. However, we cannot exclude the possibility that hydras have novel endogenous systems supporting day-night variation. For example, a recent multiomics study revealed that hundreds of genes, proteins and metabolites show robust circadian oscillations in cultured *Drosophila* S2 cells even though it is known that S2 cells do not express core clock genes [63]. Such findings clearly suggest that there is another oscillator independent of canonical clock systems.

In some cnidarians possessing orthologs of the core clock genes, it has been reported that behavioral circadian rhythms and circadian gene expression tend to dampen and then resolve within a few days under constant darkness [18] [20] [64]. A recent study also revealed that differential gene expression between midday and midnight disappears under constant darkness conditions in *Nematostella vectensis* [17]. In other words, clock systems might be not as robust in cnidarians as in other taxonomic groups, indicating that cnidarians, especially hydras*,* are intriguing subjects for understanding the evolutionary aspects of circadian clocks.

## Conclusions

In this study, we demonstrated that hydras exhibit diel rhythms in terms of behavior and gene expression despite a lack of typical clock genes under light–dark cycles. Through genome-wide transcriptome analysis, we identified 380 genes with clear diel oscillations in expression, and some of these genes shared common features with the diel cycle genes of other cnidarian species with endogenous clock systems. These results suggest that the identified genes might play an important role in inducing diel rhythms and that these types of genes are common throughout cnidarians regardless of the presence or absence of core clock genes. This is the first report of a comprehensive analysis regarding diel rhythms in hydras.

## Additional file

Additional file 1: List of *Hy*DCGs lists 380 diel cycle genes in *Hydra vulgaris*. “Gene ID” refers to the gene number registered in the Hydra 2.0 Genome Portal (https://research.nhgri.nih.gov/hydra/). The homologue descriptions and gene ontology terms are based on information in the Hydra 2.0 Genome Portal. “Cluster” represents a group of IDs determined by K-means clustering (see Fig. 4). The subsequent columns refer to the significance levels obtained from the three algorithms for rhythmicity detection (ARSER, JTK_CYCLE and empirical JTK_CYCLE) and the normalized expression levels at six time points (two biological replicates). The expression level of each gene was normalized so that the mean intensity and the standard deviation were 0.0 and 1.0, respectively. (XLS 316 kb)

## References

[1] Allada R, Emery P, Takahashi JS, Rosbash M (2001). Stopping time: the genetics of Fly and mouse circadian clocks. Annu Rev Neurosci.

[2] Tomioka K, Matsumoto A (2010). A comparative view of insect circadian clock systems. Cell Mol Life Sci.

[3] Tataroglu O, Emery P (2015). The molecular ticks of the Drosophila circadian clock. Curr Opin Insect Sci.

[4] Andreani TS, Itoh TQ, Yildirim E, Hwangbo DS, Allada R (2015). Genetics of circadian rhythms. Sleep Med Clin.

[5] Dunlap JC, Loros JJ, Liu Y, Crosthwaite SK (2001). Eukaryotic circadian systems: cycles in common. Genes Cells.

[6] Park E, Hwang DS, Lee JS, Song JI, Seo TK, Won YJ (2012). Estimation of divergence times in cnidarian evolution based on mitochondrial protein-coding genes and the fossil record. Mol Phylogenet Evol.

[7] Ball EE, Hayward DC, Saint R, Miller DJ (2004). A simple plan — cnidarians and the origins of developmental mechanisms. Nat Rev Genet.

[8] Collins AG, Schuchert P, Marques AC, Jankowski T, Medina M, Schierwater B (2006). Medusozoan phylogeny and character evolution clarified by new large and small subunit rDNA data and an assessment of the utility of phylogenetic mixture models. Syst Biol.

[9] Daly M, Brugler MR, Cartwright P, Collins AG, Dawson MN, Fautin DG, France SC, Mcfadden CS, Opresko DM, Rodriguez E, et al. The phylum Cnidaria: a review of phylogenetic patterns and diversity 300 years after Linnaeus. Zootaxa. 2007:127–82.

[10] Ji N, Flavell SW (2017). Hydra: imaging nerve nets in action. Curr Biol.

[11] Arendt D, Tosches MA, Marlow H (2016). From nerve net to nerve ring, nerve cord and brain--evolution of the nervous system. Nat Rev Neurosci.

[12] Levy O, Kaniewska P, Alon S, Eisenberg E, Karako-Lampert S, Bay LK, Reef R, Rodriguez-Lanetty M, Miller DJ, Hoegh-Guldberg O (2011). Complex diel cycles of gene expression in coral-algal symbiosis. Science.

[13] Brady AK, Snyder KA, Vize PD (2011). Circadian cycles of gene expression in the coral, Acropora millepora. PLoS One.

[14] Bertucci A, Foret S, Ball EE, Miller DJ (2015). Transcriptomic differences between day and night in Acropora millepora provide new insights into metabolite exchange and light-enhanced calcification in corals. Mol Ecol.

[15] Hemond EM, Vollmer SV (2015). Diurnal and nocturnal transcriptomic variation in the Caribbean staghorn coral, Acropora cervicornis. Mol Ecol.

[16] Oren M, Tarrant AM, Alon S, Simon-Blecher N, Elbaz I, Appelbaum L, Levy O (2015). Profiling molecular and behavioral circadian rhythms in the non-symbiotic sea anemone Nematostella vectensis. Sci Rep.

[17] Leach WB, Macrander J, Peres R, Reitzel AM (2018). Transcriptome-wide analysis of differential gene expression in response to light:dark cycles in a model cnidarian. Comp Biochem Physiol Part D: Genomics Proteomics.

[18] Reitzel AM, Behrendt L, Tarrant AM (2010). Light entrained rhythmic gene expression in the sea anemone Nematostella vectensis: the evolution of the animal circadian clock. PLoS One.

[19] Shoguchi E, Tanaka M, Shinzato C, Kawashima T, Satoh N (2013). A genome-wide survey of photoreceptor and circadian genes in the coral, Acropora digitifera. Gene.

[20] Reitzel AM, Tarrant AM, Levy O (2013). Circadian clocks in the Cnidaria: environmental entrainment, molecular regulation, and organismal outputs. Integr Comp Biol.

[21] Galliot B (2012). Hydra, a fruitful model system for 270 years. Int J Dev Biol.

[22] Chapman JA, Kirkness EF, Simakov O, Hampson SE, Mitros T, Weinmaier T, Rattei T, Balasubramanian PG, Borman J, Busam D (2010). The dynamic genome of Hydra. Nature.

[23] Passano LM, McCullough CB (1962). The light response and the rhythmic potentials of Hydra. Proc Natl Acad Sci U S A.

[24] Taddei-Ferretti C, Musio C (2000). Photobehaviour of Hydra (Cnidaria, Hydrozoa) and correlated mechanisms: a case of extraocular photosensitivity. J Photochem Photobiol B.

[25] Taddei-Ferretti C, Musio C, Santillo S: Photoresponsive behaviour in Hydra. Pflugers Archiv-European J Physiol 1998, 435:R10-R10.

[26] Guertin S, Kass-Simon G (2015). Extraocular spectral photosensitivity in the tentacles of Hydra vulgaris. Comp Biochem Physiol A Mol Integr Physiol.

[27] Passano LM, Mccullough CB (1965). Co-Ordinating systems and behaviour in Hydra .2. Rhythmic potential system. J Exp Biol.

[28] Rushforth NB, Burnett AL, Maynard R (1963). Behavior in Hydra - Contraction Responses of Hydra Pirardi to Mechanical and Light Stimuli. Science.

[29] Singer RH, Rushforth NB, Burnett AL (1963). The photodynamic action of light on Hydra. J Exp Zool.

[30] Plachetzki DC, Fong CR, Oakley TH. Cnidocyte discharge is regulated by light and opsin-mediated phototransduction. BMC Biol. 2012;10. 10.1186/1741-7007-10-17 PMC3329406 22390726

[31] Feldman M, Lenhoff HM (1960). Phototaxis in Hydra-Littoralis - rate studies and localization of the photoreceptor. Anat Rec.

[32] Badhiwala KN, Gonzales DL, Vercosa DG, Avants BW, Robinson JT (2018). Microfluidics for electrophysiology, imaging, and behavioral analysis of Hydra. Lab Chip.

[33] Schneider CA, Rasband WS, Eliceiri KW (2012). NIH image to ImageJ: 25 years of image analysis. Nat Methods.

[34] Murillo-Rincon AP, Klimovich A, Pemöller E, Taubenheim J, Mortzfeld B, Augustin R, Bosch TCG. Spontaneous body contractions are modulated by the microbiome of Hydra. Sci Rep. 2017;7. 10.1038/s41598-017-16191-x PMC5698334 29162937

[35] Bolstad BM, Irizarry RA, Astrand M, Speed TP (2003). A comparison of normalization methods for high density oligonucleotide array data based on variance and bias. Bioinformatics.

[36] Gentleman RC, Carey VJ, Bates DM, Bolstad B, Dettling M, Dudoit S, Ellis B, Gautier L, Ge Y, Gentry J (2004). Bioconductor: open software development for computational biology and bioinformatics. Genome Biol.

[37] Yang R, Su Z (2010). Analyzing circadian expression data by harmonic regression based on autoregressive spectral estimation. Bioinformatics.

[38] Hughes ME, Hogenesch JB, Kornacker K (2010). JTK_CYCLE: an efficient nonparametric algorithm for detecting rhythmic components in genome-scale data sets. J Biol Rhythm.

[39] Hutchison AL, Maienschein-Cline M, Chiang AH, Tabei SMA, Gudjonson H, Bahroos N, Allada R, Dinner AR (2015). Improved statistical methods enable greater sensitivity in rhythm detection for genome-wide data. PLoS Comput Biol.

[40] Huang DW, Sherman BT, Lempicki RA (2009). Systematic and integrative analysis of large gene lists using DAVID bioinformatics resources. Nat Protoc.

[41] Kremien M, Shavit U, Mass T, Genin A (2013). Benefit of pulsation in soft corals. Proc Natl Acad Sci U S A.

[42] Macklin M, Roma T, Drake K (1973). Water excretion by hydra. Science.

[43] Benos DJ, Prusch RD (1973). Osmoregulation in Hydra - column contraction as a function of external osmolality. Comp Biochem Physiol.

[44] Kuintzle RC, Chow ES, Westby TN, Gvakharia BO, Giebultowicz JM, Hendrix DA (2017). Circadian deep sequencing reveals stress-response genes that adopt robust rhythmic expression during aging. Nat Commun.

[45] Wu G, Zhu J, Yu J, Zhou L, Huang JZ, Zhang Z (2014). Evaluation of five methods for genome-wide circadian gene identification. J Biol Rhythm.

[46] Kass-Simon G, Pannaccione A, Pierobon P (2003). GABA and glutamate receptors are involved in modulating pacemaker activity in hydra. Comp Biochem Physiol a-Molecul Integr Physiol.

[47] Dean C, Dresbach T (2006). Neuroligins and neurexins: linking cell adhesion, synapse formation and cognitive function. Trends Neurosci.

[48] Ushkaryov YA, Petrenko AG, Geppert M, Sudhof TC (1992). Neurexins: synaptic cell surface proteins related to the alpha-latrotoxin receptor and laminin. Science.

[49] Hirokawa N, Noda Y, Tanaka Y, Niwa S (2009). Kinesin superfamily motor proteins and intracellular transport. Nat Rev Mol Cell Biol.

[50] Vale RD (2003). The molecular motor toolbox for intracellular transport. Cell.

[51] Hirokawa N, Takemura R (2005). Molecular motors and mechanisms of directional transport in neurons. Nat Rev Neurosci.

[52] Ou CY, Poon VY, Maeder CI, Watanabe S, Lehrman EK, Fu AK, Park M, Fu WY, Jorgensen EM, Ip NY, Shen K (2010). Two cyclin-dependent kinase pathways are essential for polarized trafficking of presynaptic components. Cell.

[53] Sasaki S, Shionoya A, Ishida M, Gambello MJ, Yingling J, Wynshaw-Boris A, Hirotsune S (2000). A LIS1/NUDEL/cytoplasmic dynein heavy chain complex in the developing and adult nervous system. Neuron.

[54] Niethammer M, Smith DS, Ayala R, Peng JM, Ko J, Lee MS, Morabito M, Tsai LH (2000). NUDEL is a novel Cdk5 substrate that associates with LIS1 and cytoplasmic dynein. Neuron.

[55] Black DL (2003). Mechanisms of alternative pre-messenger RNA splicing. Annu Rev Biochem.

[56] Valadkhan S (2005). snRNAs as the catalysts of pre-mRNA splicing. Curr Opin Chem Biol.

[57] Han ST, Taralova E, Dupre C, Yuste R. Comprehensive machine learning analysis of Hydra behavior reveals a stable basal behavioral repertoire. Elife. 2018;7. 10.7554/eLife.32605 PMC5922975 29589829

[58] Saunders DS. Insect clocks, third edition: Elsevier Science; 2002.

[59] Takahashi T, Hayakawa E, Koizumi O, Fujisawa T (2008). Neuropeptides and their functions in Hydra. Acta Biol Hung.

[60] Bosch TCG, Klimovich A, Domazet-Loso T, Grunder S, Holstein TW, Jekely G, Miller DJ, Murillo-Rincon AP, Rentzsch F, Richards GS (2017). Back to the basics: cnidarians start to fire. Trends Neurosci.

[61] Ruggieri RD, Pierobon P, Kass-Simon G (2004). Pacemaker activity in hydra is modulated by glycine receptor ligands. Comp Biochemi Physiol a-Molecul Integr Physiol.

[62] Marina invertebrate models database. http://marimba.obs-vlfr.fr/home. Accessed 28 Nov 2018.

[63] Rey G, Milev NB, Valekunja UK, Ch R, Ray S, Silva Dos Santos M, Nagy AD, Antrobus R, MacRae JI, Reddy AB (2018). Metabolic oscillations on the circadian time scale in Drosophila cells lacking clock genes. Mol Syst Biol.

[64] Hoadley KD, Szmant AM, Pyott SJ (2011). Circadian clock gene expression in the coral Favia fragum over diel and lunar reproductive cycles. PLoS One.

[65] Hydra 2.0 genome Project Portal. https://research.nhgri.nih.gov/hydra/. Accessed 14 June 2018.

[66] Joint Genome Institute data base. https://genome.jgi.doe.gov/Nemve1/Nemve1.info.html. Accessed 19 Oct 2018.

[67] Putnam NH, Srivastava M, Hellsten U, Dirks B, Chapman J, Salamov A, Terry A, Shapiro H, Lindquist E, Kapitonov VV (2007). Sea anemone genome reveals ancestral eumetazoan gene repertoire and genomic organization. Science.

